# Prevalence of Alzheimer's Disease and Parkinson's Disease in China: An Updated Systematical Analysis

**DOI:** 10.3389/fnagi.2020.603854

**Published:** 2020-12-21

**Authors:** Lei Cui, Na-Na Hou, Hong-Mei Wu, Xiang Zuo, Yi-Zhi Lian, Chao-Nan Zhang, Zhen-Feng Wang, Xiong Zhang, Jian-Hong Zhu

**Affiliations:** ^1^Department of Geriatrics and Neurology, The Second Affiliated Hospital and Yuying Children's Hospital, Wenzhou Medical University, Wenzhou, China; ^2^Department of Preventive Medicine, Wenzhou Medical University, Wenzhou, China; ^3^Shanghai 9th People's Hospital, Shanghai Jiao Tong University School of Medicine, Shanghai, China

**Keywords:** Alzheimer's disease, Parkinson's disease, prevalence, epidemiology, China, systematical analysis

## Abstract

**Background:** Alzheimer's disease (AD) and Parkinson's disease (PD) are two major neurodegenerative diseases worldwide. Demographic aging is in rapid progress in China. Up-to-date estimates of AD and PD prevalence have not been provided.

**Methods:** Studies that reported the prevalence of AD and PD in China were identified via a systematic database search from 1985 to 2018. Meta-analysis, local polynomial regression and autoregressive integrated moving average model were used for analyses.

**Results:** A total of 99 studies were included in the study with populations of 385,312 and 227,228, respectively for AD and PD. The overall prevalence of AD and PD following age standardization was 3.20% [95% confidence interval (CI) = 3.17–3.23] and 1.06% (95% CI = 1.02–1.10), respectively in individuals over 60 years old. The rates increased drastically for every 10-years increment of age. The yearly prevalence of AD was predicted to increase from 3.81 to 6.17% in the next 5 years. Significant differences were observed between genders [male to female odds ratio (OR) for AD = 0.57, 95% CI = 0.51–0.64; OR for PD = 1.25, 95% CI = 1.06–1.46], and between education levels (Illiterate to non-illiterate OR for AD = 2.99, 95% CI = 2.38–3.75), but not between urban and rural settings.

**Conclusion:** Our results provide an updated insight into the epidemiology of AD and PD in China and their associated rates and ratios. The findings may facilitate China policy makers and health professionals mitigate the related health issues.

## Introduction

Alzheimer's disease (AD) and Parkinson's disease (PD) are the two most common neurodegenerative diseases affecting millions of people worldwide. AD is clinically manifested by progressive impairment in cognition, learning ability, memory function, and executive reasoning (Jiang et al., 2016). PD is characterized by motor symptoms, including bradykinesia plus rigidity and resting tremor, as well as postural instability at a more advance stage. They may also be coupled with non-motor features, such as dementia, depression, and autonomic dysfunctions (Kalia and Lang, 2015). Both AD and PD include a long asymptomatic prodromal period. At the time of diagnosis, extensive and irreversible damages have occurred in the patients (Villemagne et al., 2013; Kalia and Lang, 2015). Drugs and/or surgery are available for symptomatic relief, but thus far nothing is available yet to delay or reverse disease progression. Given their increasingly enlarged size and lack of therapeutics, AD and PD have become two of the leading causes of disability, which greatly burdens patients and their families, as well as social health and care systems (Rajiah et al., 2017; Henderson et al., 2019).

It was predicted in 2006 that China will have 248 million elderly people by 2020, accounting for 17.2% of the total population (Chow, 2006). This aged size indicates a high popularity of AD and PD patients in China since aging is the greatest risk factor for both degenerations (Morens et al., 1996; Wyss-Coray, 2016; Collier et al., 2017). Due to their adverseness and heavy burdens as mentioned above, it is of great importance to understand the size and distribution of AD and PD for clinicians and policy makers guiding health and care services.

Current messages of prevalence of AD and PD in China are fragmental. The studies were mostly restricted into specific geographic locations with various size of populations as summarized afterwards. Indications of the country's prevalence rates were dated back to decades ago for both diseases (Li et al., 1985; Xue et al., 1997). Thus, an updated understanding of the prevalence of AD and PD in China and their longitudinal, demographic, and geographic characteristics is in great demand.

## Methods

### Literature Search

A parallel systematic review of published literature from 1985 to 2018 was performed using PubMed and three Chinese databases including WanFang, VIP and China National Knowledge Infrastructure (CNKI). The search keywords included “Parkinson” or “Parkinson's,” “Alzheimer” or “Alzheimer's,” “prevalence,” “epidemiology” or “survey,” and “China” or “Chinese” (in Chinese for Chinese literature). If needed, the references were reviewed to identify potential eligible studies. The study acquired all of its data from the literature, and thus ethical approval was not required.

### Selection Criteria

The inclusion criteria were as follows: population-based studies; AD or PD studies with Chinese people over the age of 60; cross-sectional studies providing the prevalence rates of AD or PD; clear diagnostic criteria. The exclusion criteria were: duplicate publication (in this case more comprehensive, detailed, and reliable sets were selected); overlapped data in different studies; studies of case-control, hospital-based, elderly social welfare homes, and nursing homes; unclear or non-conventional diagnostic criteria.

### Data Extraction and Literature Quality Evaluation

The authors N.-N. Hou and X. Zuo completed literature retrieval and selection independently. When there was a disagreement, L. Cui and H.-M. Wu participated to reach a consensus. Extracted from the studies were author name, publication year, gender, age range, study design, response rate, diagnostic criteria, case number, study location, urban/rural, education, and sample size. All eligible studies were systematically evaluated for quality based on their sample size, study design, response rate and diagnostic assessment. The detailed scoring criteria were performed as previously described by Prince et al. (2013).

### Data Conversion

The prevalence rates of AD and PD were converted for normality by proportion, logit/log transformation, arcsine/double arcsine transformation, or Freeman-Tukey. When censoring age groups, the age range, such as ≥80 years was used as an identical range of other age groups, such as 80–85. If the case number was “0” for a certain group, “0.001” was used to prevent data from being excluded from calculations. These conversions were performed using R version 3.3.0 (R Foundation for Statistical Computing, Vienna, Austria). Age standardization was calculated from the 2010 Chinese census by the direct method.

### Statistical Analysis

Q-statistics and I^2^ were used for assessing the heterogeneity. A DeSimonian and Laird random effects model was applied when heterogeneity was found by Q-statistics (*p* < 0.1) or when I^2^ > 50%. A Mantel-Haenszel fixed-effects model was applied otherwise. Local polynomial regression was used to observe the distribution and trend of the prevalence in each age group. An autoregressive integrated moving average model was used to fit the pooled prevalence from 1997 to 2018, and to identify the optimal method based on either the largest value of the coefficient of determination (R^2^) or the lowest value of the normalized Bayesian Information Criterion (BIC). The mimic and predictive results were given by the best fitting method. An Ljung-Box Q test was adopted to diagnose whether the estimated residuals met the demand of a white-noise series. The above analyses were performed using Statistical Product and Service Solutions (SPSS; version 22.0) for windows. The online software of Dituhui (http://www.dituhui.com/) was used to construct the geographical distribution of prevalence in China. A two-tailed *p* value < 0.05 was considered statistically significant.

## Results

### Systematic Review and Record Identification

**Figure 1:**
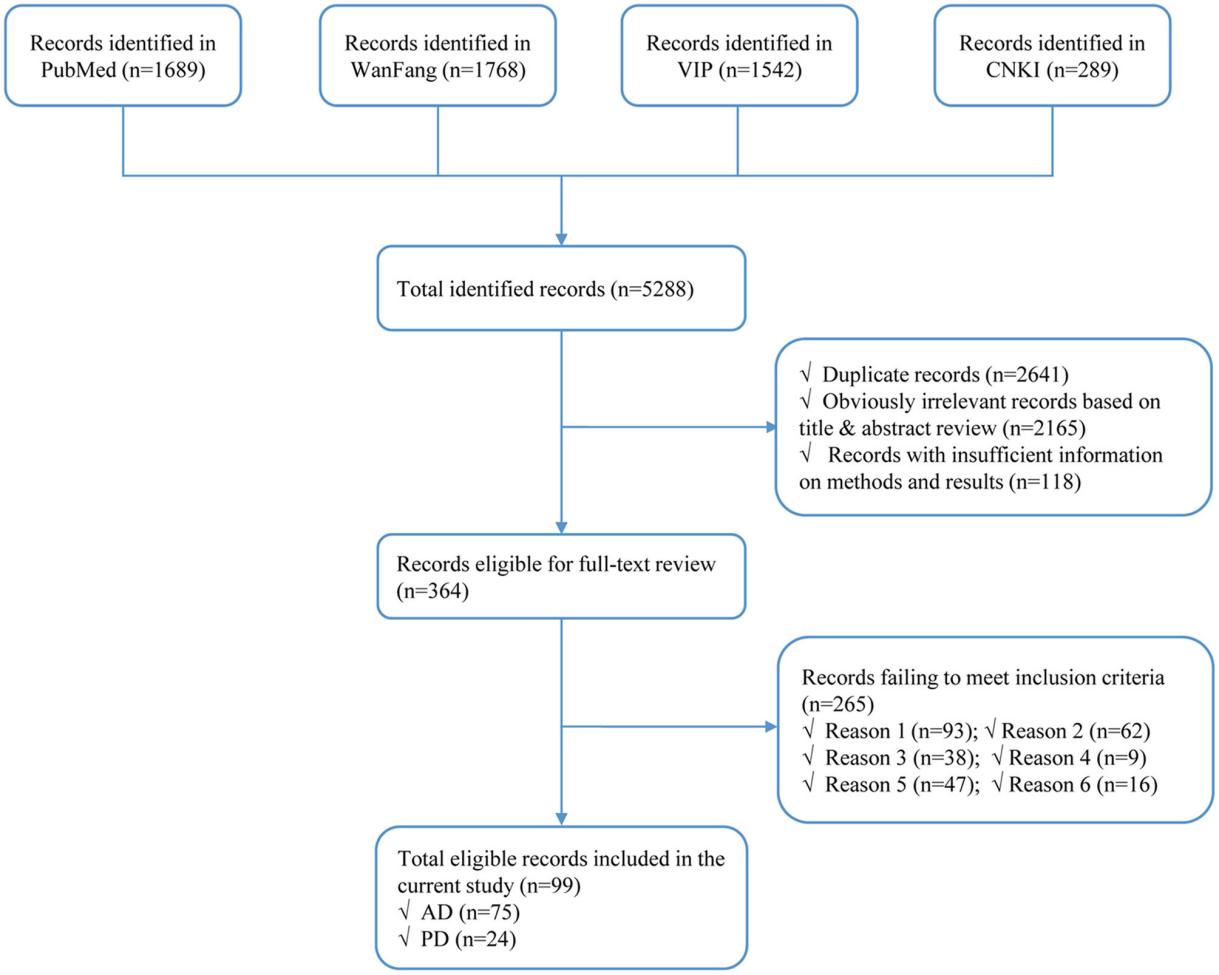
Flow diagram of study identification. AD, Alzheimer's disease. PD, Parkinson's disease; Reason 1, not population-based; Reason 2, not based in China; Reason 3, no numerical prevalence measurement; Reason 4, conducted in an unrepresentative population, such as hospital-based, in elderly social welfare, etc.; Reason 5, overlapped data in different studies; Reason 6, unclear diagnosis.

Our initial search identified a total of 5,528 citations from PubMed, WanFang, VIP, and CNKI databases. After elimination of duplicates and records of irrelevancy or insufficient information, 364 records remained. After further full-text review, 265 records were excluded based on the inclusion and exclusion criteria (Figure 1). Thus, a total of 99 records were eligible for data extraction, including 75 records for AD (Supplementary Table 1) and 24 for PD (Supplementary Table 2). The information of the included studies was described in Supplementary Tables 1, 2, including location, gender, setting, education, phase design, response rate, diagnostic assessment, diagnosis criteria, age, sample size, and quality score.

### Age-Associated Prevalence of AD and PD in China

**Figure 2:**
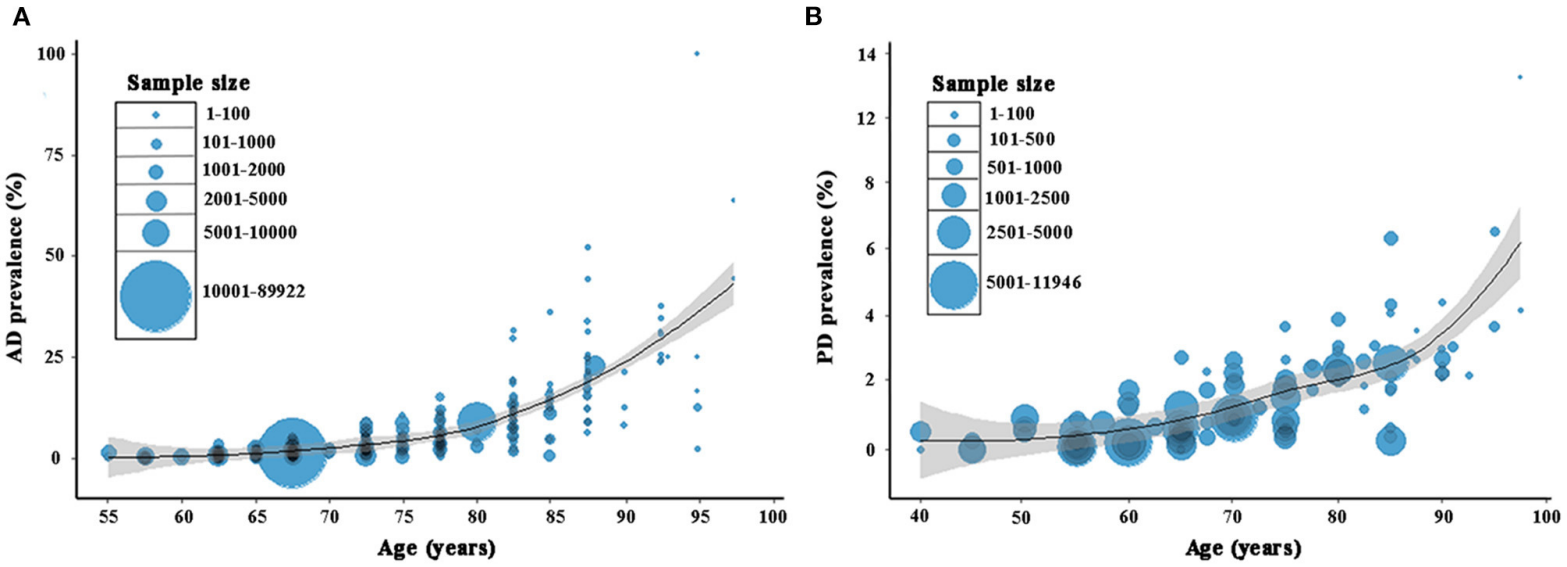
Age-dependent prevalence of AD **(A)** and PD **(B)** in China. A total of 285 and 103 data points were used, respectively for AD and PD based on the included studies. AD, Alzheimer's disease. PD, Parkinson's disease.

Based on the acquired 285 data points from AD studies and 103 data points from PD studies, the estimation of age-associated prevalence rates was constructed. The rates of both AD and PD increased steadily with advances in age, but this was more pronounced for AD (Figure 2). In specific, the rate of AD was 2.99% (95% CI = 2.52–3.49; Supplementary Figure 1A) and of PD was 1.11% (95% CI = 0.78–1.57; Supplementary Figure 1B) in individuals over 60 years old (Table 1). Further analysis showed that the prevalence rate of AD ranged from 1.03% (95% CI = 0.77–1.33) in 60–69 years old to 12.04% (95% CI = 10.24–13.96) above 80 years old. In contrast, the prevalence of PD was much lower, ranging from 0.62% (95% CI = 0.37–0.92) in 60–69 years old to 2.21% (95% CI = 1.30–3.34) above 80 years old. Following age standardization, the prevalence rates of AD and PD in people aged over 60 years were 3.20% (95% CI = 3.17–3.23) and 1.06% (95% CI = 1.02–1.10), respectively (Table 1).

**Table 1:** Prevalence of AD and PD in different ages and years in China.

	**Age/Time**	**Studies, *n***	**Cases, *n***	**Population, *n***	**Prevalence (95% CI), %**	***P-*value^a^**
AD	**Age (y)**					
	60–69	39	784	67,372	1.03 (0.77–1.33)	
	70–79	48	2,377	61,789	3.75 (3.08–4.49)	
	≥80	50	2,560	21,993	12.04 (10.24–13.96)	
	≥60	52	10,316	311,293	2.99 (2.52–3.49)	
	≥60^b^	51	16,037	462,447	3.20 (3.17–3.23)	
	**Time period**					
	1997–2001	8	314	17,280	1.68 (1.08–2.62)	
	2002–2006	13	1,316	75,266	1.90 (1.21–2.73)	0.5360
	2007–2011	15	1,518	41,738	3.65 (2.96–4.39)	<0.0001
	2012–2016	13	6,757	166,578	4.16 (3.42–4.90)	<0.0001
	2017–2018	3	411	10,431	3.96 (2.30–6.06)	0.5593
PD	**Age (y)**					
	60–69	12	210	33,084	0.62 (0.37–0.92)	
	70–79	14	336	23,641	1.41 (1.02–1.88)	
	≥80	16	354	16,523	2.21 (1.30–3.34)	
	≥60	14	836	73,966	1.11 (0.78–1.57)	
	≥60^b^	17	900	73,248	1.06 (1.02–1.10)	
	**Time period**					
	1985–1999	3	59	9,590	0.94 (0.24–3.67)	
	2000–2014	8	682	55,479	1.14 (0.72–1.64)	0.0301
	2015–2018	3	95	8,897	2.04 (0.00–4.13)	0.0169

a *Calculated by comparing to the previous period*.

b *Standardized with age calculated from the 2010 Chinese population census by use of the direct method*.

*AD, Alzheimer's disease; CI, confidence interval; PD, Parkinson's disease; y, years old*.

We also performed an analysis based on diagnostic criteria. A significant heterogeneity was detected among the studies under the same diagnostic criterion (Supplementary Table 3). A random effects model was then used for meta-analysis. The prevalence of AD and PD appeared to be variable among different diagnostic criteria. The prevalence of AD was highest in the subgroup of mixed criteria (4.51%), and lowest in the subgroup of DSM-III-R (1.01%). The prevalence of PD was higher in the subgroup of UK Parkinson's disease Society Brain Bank Criteria than that of other criteria (1.71 vs. 0.99%; Supplementary Table 3).

### Prevalence of AD and PD by Year in Individuals Over 60 Years Old

The pooled prevalence rates of AD in 1997–2001, 2002–2006, 2007–2011, 2012–2016, and 2017–2018 were 1.68, 1.90, 3.65, 4.16, and 3.96%, respectively, with a significant (*P* < 0.05) increase in 2007–2011 and 2012–2016 compared to its respective previous period. The pooled prevalence rates of PD in 1985–1999, 2000–2014 and 2015–2018 were 0.94, 1.14, and 2.04%, respectively, with a significant (*P* < 0.05) increase in the latter two periods compared to its respective previous period (Table 1).

**Figure 3:**
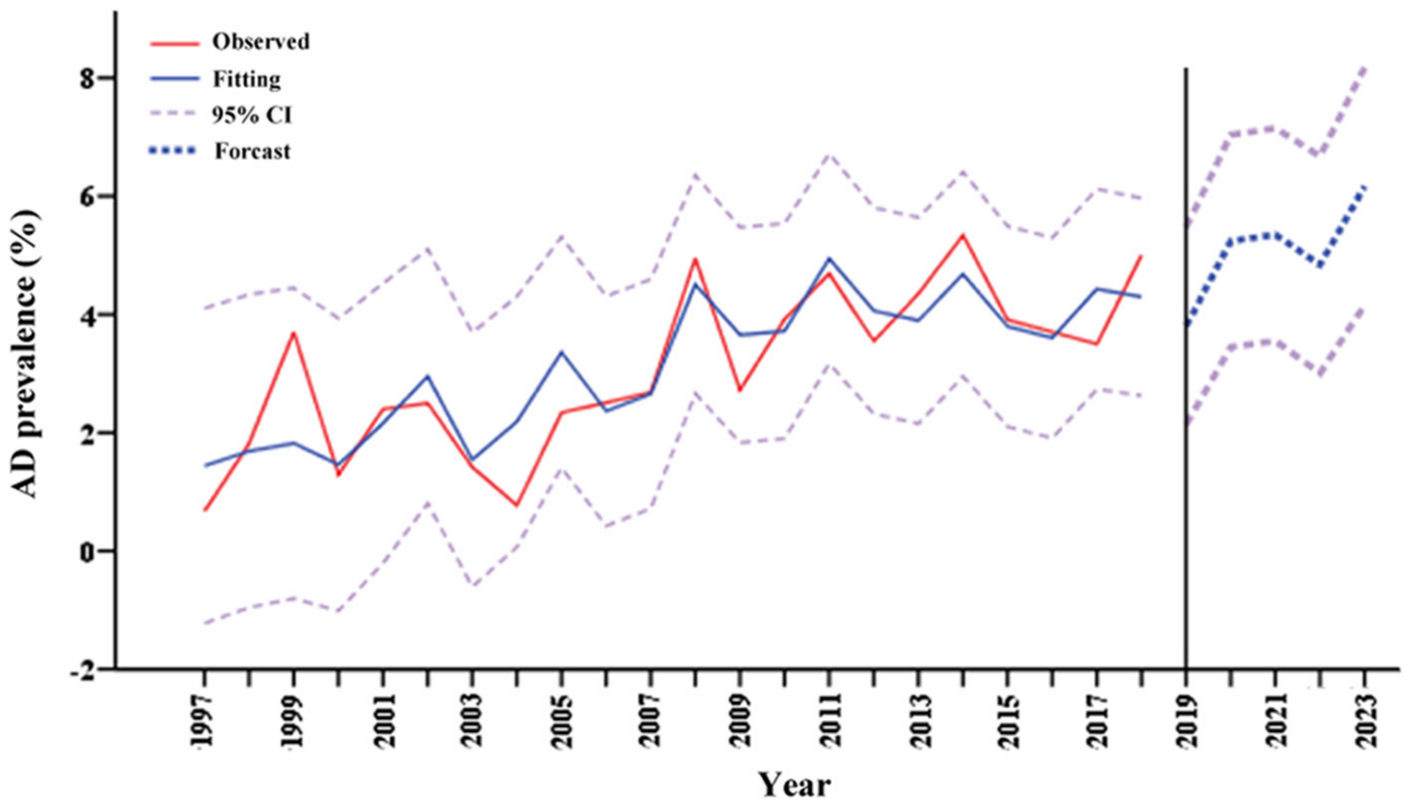
Prediction of AD prevalence in Chinese over 60 years old in the next 5 years. AD, Alzheimer's disease.

Based on the yearly pooled prevalence of AD from 1997 to 2018 in China, a model (4, 0, 4) was identified as the best fitting specification (R^2^ = 0.724, normalized BIC = 1.316, *P* > 0.05 for Ljung-Box Q test). The rate of AD showed a slightly increasing trend with no seasonal variation, and was predicted to be 3.81% (95% CI = 2.14–5.47), 5.24% (95% CI = 3.45–7.04), 5.35% (95% CI = 3.55–7.15), 4.84% (95% CI = 3.01–6.68), and 6.17% (95% CI = 4.16–8.18) for the next 5 years from 2019 to 2023 (Figure 3). PD was not analyzed due to insufficient data.

### Prevalence of AD and PD by Gender and Education

The prevalence rates of AD and PD for men were 2.30% (95% CI = 1.86–2.78) and 1.20% (95% CI = 0.76–1.73), respectively and for women were 4.17% (95% CI = 3.56–4.78) and 0.87% (95% CI = 0.56–1.24), respectively. A significant difference was found between the genders for both AD and PD (men compared to women, AD: OR = 0.57, 95% CI = 0.51–0.64; PD: OR = 1.25, 95% CI = 1.06–1.46; Table 2, Supplementary Figures 2A,B).

**Table 2:** Effect of gender, education and setting on AD and PD prevalence.

	**Characteristics**	**Studies, *n***	**Cases, *n***	**Population, *n***	**Prevalence (95% CI), %**	***P-*value^a^**	**OR (95% CI)**
AD	**Gender**						
	Male	50	2,372	97,160	2.30 (1.86–2.78)	<0.0001	0.57 (0.51–0.64)
	Female	50	3,956	105,325	4.17 (3.56–4.78)		
	**Education**						
	Illiterate	35	2,216	47,926	5.39 (4.28–6.61)	<0.0001	2.99 (2.38–3.75)
	Non-illiterate	35	2,046	87,029	1.92 (1.50–2.39)		
	**Setting**						
	Urban	14	939	34,190	3.14 (2.53–3.88)	0.1102	0.84 (0.68–1.04)
	Rural	14	934	27,423	3.92 (2.85–5.15)		
PD	**Gender**						
	Male	19	845	84,477	1.20 (0.76–1.73)	0.0074	1.25 (1.06–1.46)
	Female	19	609	61,884	0.87 (0.56–1.24)		

a *Calculated by comparing to its respective counterpart*.

*AD, Alzheimer's disease; CI, confidence interval; PD, Parkinson's disease*.

In the context of education, the prevalence of AD was 5.39% (95% CI = 4.28–6.61) for the illiterate and 1.92% (95% CI = 1.50–2.39) for the non-illiterate. The education showed a significant impact on the prevalence rate (OR = 2.99, 95% CI = 2.38–3.75; Table 2; Supplementary Figure 3A). PD was not analyzed due to insufficient data.

### Prevalence of AD and PD by Setting and Geography

The effect of setting was calculated only for AD. The prevalence rates were 3.14% (95% CI = 2.53–3.88) for the urban population and 3.92% (95% CI = 2.85–5.15) for the rural population. There was no significant difference between the settings (Table 2; Supplementary Figure 3B).

**Figure 4:**
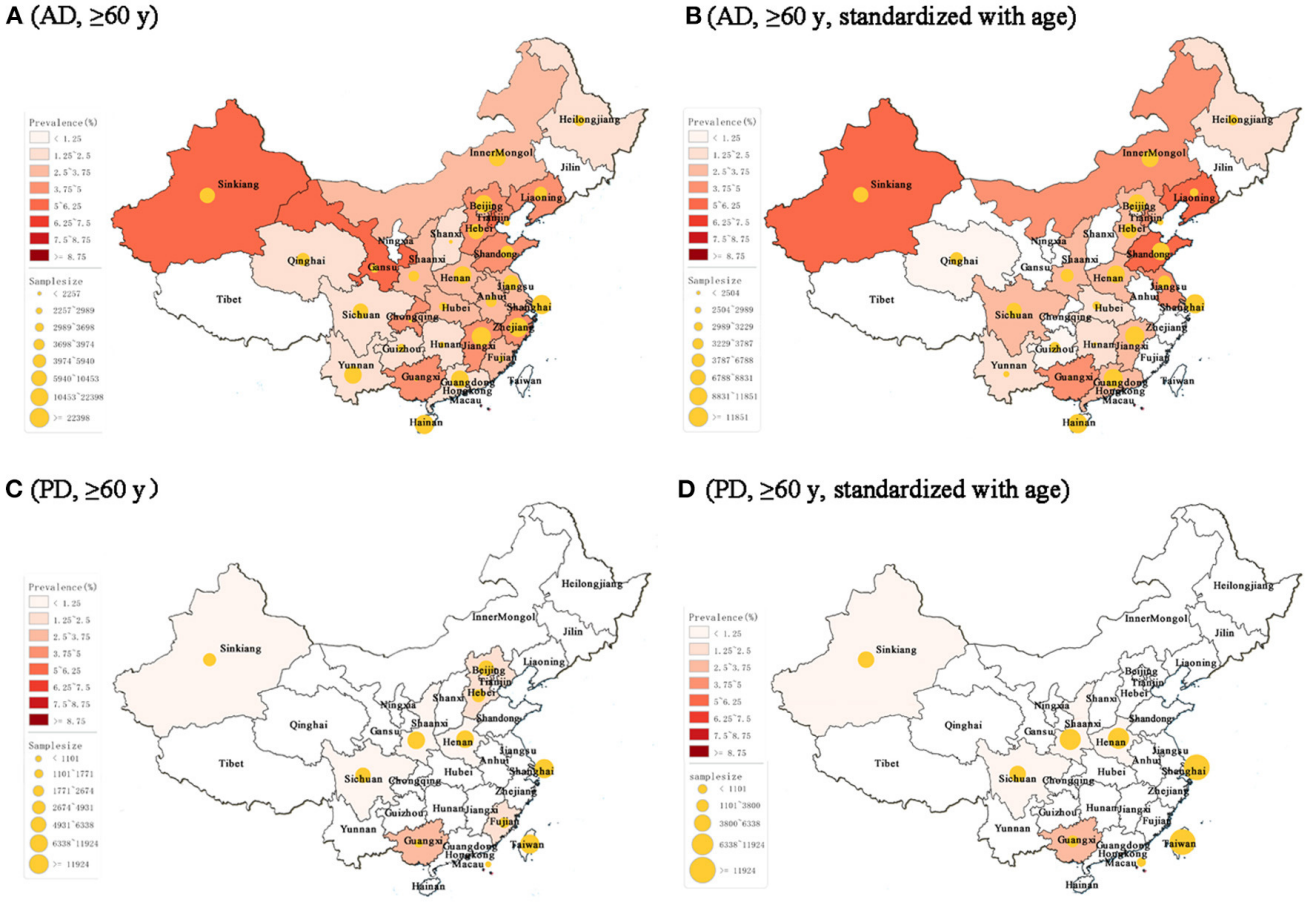
Spatial distribution of AD and PD prevalence rates across China in populations over 60 years old. **(A)** The prevalence of AD. As a note, the age for Anhui, Gansu, and Chongqing was over 65 years. **(B)** Age-standardized prevalence of AD. **(C)** The prevalence of PD. As a note, the age for Hebei and Fujian was over 65 years. **(D)** Age-standardized prevalence of PD. AD, Alzheimer's disease. PD, Parkinson's disease; y, years old.

Spatial distribution maps of prevalence of AD and PD over 60 years old were constructed from individual provinces across China (Figure 4). According to the geographical characteristics of China, the study locations were classified into seven geographic regions: East China, North China, Northeast China, Northwest China, South Central China, Central China, and Southwest China (Supplementary Table 4). Varied pooled prevalence rates were present in different geographic regions in China (Table 3). The prevalence of AD was of the highest in Northwest China (4.36%, 95% CI = 2.59–6.13), and the lowest in South China (1.26%, 95% CI = 0.58–2.71). The prevalence of PD was of the highest in South China (3.25%, 95% CI = 2.45–4.15), and the lowest in Southwest China (0.34%, 95% CI = 0.18–0.51). Following standardization with the population of the respective regions, the overall pooled prevalence of AD and PD was 2.90% (95% CI = 2.84–2.96) and 1.20% (95% CI = 1.12–1.29), respectively (Table 3).

**Table 3:** Prevalence of AD and PD in different geographic regions of China.

**Geographic region**	**AD**			**PD**		
	**Prevalence (95% CI), %**	**Studies, *n***	**Population, *n***	**Prevalence (95% CI), %**	**Studies, *n***	**Population, *n***
North China	3.72 (2.78–4.67)	14	36,495	2.15 (1.74–2.66)	3	7,605
Northeast China	3.45 (2.10–5.67)	4	8,848	–	–	–
East China	3.55 (2.96–4.15)	18	182,448	1.12 (0.83–1.44)	5	27,438
South China	1.26 (0.58–2.71)	7	34,526	3.25 (2.45–4.15)	2	1,662
Central China	2.26 (1.53–2.99)	3	15,897	0.74 (0.58–0.90)	1	10,651
Southwest China	1.94 (1.20–3.14)	5	22,455	0.34 (0.18–0.51)	1	4,944
Northwest China	4.36 (2.59–6.13)	6	19,042	0.61 (0.12–1.46)	2	10,138
All^a^	2.90 (2.84–2.96)	57	319,711	1.20 (1.12–1.29)	14	62,438

a *Standardized with regional population calculated from the 2010 Chinese population census by use of the direct method*.

*AD, Alzheimer's disease; CI, confidence interval; PD, Parkinson's disease*.

## Discussion

As the two top-ranked neurodegenerative diseases, AD and PD place an increasing burden on the already stretched health care services in China together with an aging demography and a lack of effectiveness in prevention, detection, and treatment of the diseases (Dubois et al., 2016; Rizek et al., 2016). The current study represents an updated systematic analysis to understand the national and subnational prevalence rates and risk factors of AD and PD in China. A wide geographical covering of the included studies ensures a sufficient power for the estimates of prevalence.

As suggested in the study, the age-standardized prevalence rates of AD and PD in individuals aged over 60 years are 3.20 and 1.06%, respectively in China, and are approximately comparable to those standardized with the regional population (that is, 2.90 and 1.20%, respectively). These numbers are also comparable to those which previously reported that the prevalence of AD was 3.21% in China (Jia et al., 2014), and that PD affected 1.1% of people over 60 years old worldwide (Pringsheim et al., 2014). On the contrary, there are also studies reporting higher prevalence of AD, such as in the United States (5.7%) (Steenland et al., 2016), Europe (5.05%) (Niu et al., 2017), and other Asian countries including Korea (5.7%) (Kim et al., 2011) and Japan (3.8%) (Sekita et al., 2010). These global differences may attribute to environmental risks, genetic factors, lifestyles and study methodologies (Catindig et al., 2012; Prince et al., 2013). In addition, the current prevalence of PD is slightly lower than that reported previously in China (1.7%) (Zhang et al., 2005) and Korea (1.47%) (Seo et al., 2007), which may result from disparities in time period, geographical distribution, and diagnostic criterion across individual studies (Pringsheim et al., 2014). Indeed, the rate of prevalence can be affected by diagnostic criteria as shown in the current study, indicating an importance of consistency in disease evaluation methodology. Although the estimation of AD prevalence is shown to elevate in the next 5 years, the already-occurred rates of AD and PD appear to remain steady up to 2018.

As expected, age is the most important risk factor for both diseases. The prevalence rate of AD is increased by ~11.7 times in individuals from 60–69 years old to above 80 years old. The increase for PD is about 2.6-folds. In line with these results, previous studies suggested an increase of AD prevalence from 0.97% in people between ages of 65 and 74 to nearly 14.4% in those over the age of 80 (Gao et al., 1998), and an increase of PD rates from 0.43% between ages of 60 and 70 to 1.9% in those over the age of 80 (Pringsheim et al., 2014). As well-recognized, there is a gender effect for both AD and PD. It has been noted that the male-to-female ratio of AD is 0.473 in European (Niu et al., 2017), and of PD is averagely 1.5 worldwide (De Lau and Breteler, 2006). Our results demonstrate that the male-to-female ratio is 0.57 for AD and 1.25 for PD in China.

There is no difference in the prevalence rates of AD between rural and urban areas in China. A comment published by the World Neurology Dementia Research Group showed that the prevalence of AD was different between urban and rural areas in Latin America, but not in Africa, and the situation in developed regions and Asia remained unclear (Members et al., 2010; Chan et al., 2013). Meanwhile, we find that higher education is a protective factor against AD, which is in line with most of the previous results (Ott et al., 1995; Obadia et al., 1997; Kalaria et al., 2008; Bickel and Kurz, 2009; Members et al., 2010).

The regional subgroup analyses suggest a large variation of prevalence rates in both AD and PD in China. People living in northwestern China are more likely to develop AD, which is about 2.5 times higher than the lowest South China region. In contrast, the highest region of PD prevalence is South China, about 8.6 times higher than its lowest region, Southwest China. A similar trend of AD is also indicated in a previous study, which showed that the prevalence of dementia was highest in the west, followed by the north, central and south of China after adjusting for methodological variation (Wu et al., 2018). However, the covering of geographic regions for PD is limited, cautioning less reliability. These variations may be attributed to differences in geographical environment and ethnicity for some areas. For example, levels of sunlight exposure and vitamin D vary with geographic latitude and are found to be associated with AD in older adults (Jayedi et al., 2019). Air pollution, an environmental risk factor for both AD and PD (Fu and Yung, 2020; Salimi et al., 2020), is much more severe in the north and northwest regions of China (Kuerban et al., 2020). Ethnicity also varies across China, such as Han in most regions, Uyghur, and Tibetans mostly in the northwest and west regions, respectively. In addition, education, smoking, nutrition, and dietary pattern across the life course have been presented as contributors to the prevalence of AD and PD and provide additional explanations for the geographical variation (Wu et al., 2018; Kuerban et al., 2020).

There are multiple limitations rendering a caution in interpreting the results. First, given the diversity in study designs, targeted populations, methods, and settings, a relatively high degree of heterogeneity among the included studies was observed. Second, a potential uncertainty in sensitivity and specificity of the case diagnosis may exist in the cross-sectional assessments, which however could not be completely controlled. Third, despite our extensive effort to identify available evidence, the number of eligible studies for PD prevalence is still not sufficient, which hampers further subgroup analyses and renders a potential lack of sufficient power. Fourth, due to the limited eligible studies, the sample size in our study is relatively small for the total population of China to estimate the prevalence of AD and PD.

In conclusion, this contemporary systematic review and analysis provides an updated insight into the epidemiology of AD and PD in China and their associated variations in the context of age, gender, education, setting, and geographic region. The findings may facilitate China policy makers and health professionals in optimizing public health strategies and mitigating the AD and PD associated health issues.

## Data Availability Statement

The original contributions presented in the study are included in the article/Supplementary Material, further inquiries can be directed to the corresponding author/s.

## Author Contributions

J-HZ and XZh conceived the idea and designed the research. LC, N-NH, H-MW, and XZu performed the data collection, extraction, and analyses. Y-ZL and C-NZ contributed to the data verification. Z-FW contributed to the map construction. N-NH, LC, and J-HZ wrote the manuscript. All authors read, reviewed, and approved the final manuscript.

## Conflict of Interest

The authors declare that the research was conducted in the absence of any commercial or financial relationships that could be construed as a potential conflict of interest.
