# Supplementary material for: Prevalence of Alzheimer's Disease and Parkinson's Disease in China: An Updated Systematical Analysis
Source: Front Aging Neurosci. 2020 Dec 21;12:603854. doi: 10.3389/fnagi.2020.603854 (PMC7793643; doi:10.3389/fnagi.2020.603854)
Supplement: Supplementary file 1 [file Table_1.DOC]

**Table S1.** Detailed characteristics of 75 AD studies

| Study | Location | Gender | Setting | Education | Phase design | Response rate (%) | Screening | Diagnosis | Age range | Sample  size | Cases | Quality score |
| --- | --- | --- | --- | --- | --- | --- | --- | --- | --- | --- | --- | --- |
| Wang et al., 1999^[1]^ | Anhui | Mixed | Both | No | two | 92.03 | HDS | NINCDS-ADRDA;  ICD-10;  CCMD-Ⅱ-R | 65+ | 2749 | 145 | 8.5 |
| Li et al., 2011^[2]^ | Anhui | Both | Rural | Yes | two | 88.30 | CSI-D;  GMSA | CCMD-Ⅲ | 65+ | 1075 | 21 | 8 |
| Xing et al., 2018^[3]^ | Beijing | Mixed | Both | Yes | three | 99.52 | ADL;  MMSE;  interview | DSM-IV | 60+ | 2259 | 113 | 8.5 |
| Tang et al., 2002^[4]^ | Beijing | Both | Both | Yes | two | 81.60 | MMSE;  interview | DSM-IV;  NINCDS-ADRDA | 60+ | 2788 | 140 | 9.5 |
| Xie et al., 2000^[5]^ | Beijing | Mixed | NA | No | two | 88.59 | MMSE;  interview | DSM-Ⅲ-R;  NINCDS-ADRDA | 60+ | 1491 | 26 | 8 |
| Cui et al., 2012^[6]^ | Beijing | Both | NA | Yes | three | 91.30 | MMSE;  MoCA;  ADL;  interview | DSM-IV;  NINCDS-ADRDA | 60+ | 3473 | 117 | 10 |
| Li et al., 1999^[7]^ | Beijing | Both | Rural | No | two | 90.30 | MMSE | DSM-Ⅲ-R | 60+ | 1027 | 38 | 7 |
| Zhang et al., 1998^[8]^ | Beijing | Both | NA | Yes | two | 89.42 | MMSE | DSM-Ⅲ-R | 60+ | 1243 | 17 | 7 |
| Zheng et al., 2010^[9]^ | Beijing | Mixed | Both | Yes | two | 100.00 | MMSE;  HIS;  interview | ICD-10 | 60+ | 1756 | 57 | 10.5 |
| Chen et al., 2009^[10]^ | Fujian | Both | Urban | No | two | 96.42 | MMSE;  ADL;  interview | DSM-IV;  NINCDS-ADRDA | 60+ | 2373 | 81 | 9.5 |
| Li et al., 2009^[11]^ | Fujian | Both | Rural | No | two | 92.6 | MMSE;  ADL;  interview | DSM-IV | 65+ | 2696 | 134 | 9.5 |
| Zhang et al., 2017^[12]^ | Gansu | Both | NA | No | two | 100.00 | MMSE;  interview | CDR;  HIS | 55+ | 2242 | 64 | 8.5 |
| Wang et al., 2016^[13]^ | Gansu | Both | Both | No | two | 99.42 | HDS;  HIS;  CDR;  interview | DSM-IV | 65+ | 1859 | 108 | 10.5 |
| Huang et al., 2014^[14]^ | Guangdong | Both | NA | Yes | two | 100.00 | interview | NINCDS-ADRDA | 65+ | 3368 | 177 | 8 |
| Liang et al., 2003^[15]^ | Guangdong | Both | NA | Yes | two | 89.58 | MMSE;  HDS;  ADL | DSM-IV;  NINCDS-ADRDA | 60+ | 1418 | 51 | 9 |
| Xue et al., 1997^[16]^ | Guangdong | Mixed | Mixed | No | two | 77.09 | MMSE;  interview | DSM-Ⅲ-R;  NINCDS-ADRDA | 60+ | 3285 | 22 | 8 |
| Tang et al., 2007^[17]^ | Guangdong | Both | Urban | No | two | 93.20 | MMSE;  ADL;  CES-D; interview | DSM-IV;  NINCDS-ADRDA | 55+ | 5276 | 128 | 11 |
| Ma et al., 2005^[18]^ | Guangdong | Mixed | Both | No | two | 96.43 | MMSE;  ADL;  CES-D; interview | DSM-IV;  NINCDS-ADRDA | 65+ | 3780 | 128 | 11 |
| Lai et al., 2000^[19]^ | Guangdong | Mixed | Urban | No | three | 80.50 | MMSE;  ADL; | DSM-Ⅲ-R;  NINCDS -ADRDA | 75+ | 3285 | 190 | 11 |
| Gao et al., 2004^[20]^ | Guangdong | Both | NA | No | two | 93.54 | MMSE;  interview | DSM-IV-R;  NINCDS-ADRD | 60+ | 1839 | 22 | 8 |
| Yu et al., 1998^[21]^ | Guangdong | Both | Rural | No | two | 98.74 | HDS | ICD-10;  CCMD-Ⅱ-R | 65+ | 1018 | 48 | 8 |
| Li et al., 2015^[22]^ | Guangxi | Both | NA | Yes | two | 79.40 | MMSE;  ADL; | DSM-IV;  NINCDS-ADRDA | 60+ | 889 | 38 | 8 |
| Huang et al., 2007^[23]^ | Guizhou | Mixed | Mixed | Yes | two | 81.50 | MMSE;  ADL;  interview | DSM-IV;  NINCDS-ADRDA | 60+ | 3229 | 41 | 10 |
| Wu et al., 2003^[24]^ | Hainan | Mixed | NA | No | two | 100.00 | MMSE | DSM-Ⅲ-R | 60+ | 9770 | 28 | 8 |
| Chen et al., 2004^[25]^ | Hainan | Both | NA | No | two | 100.00 | MMSE | DSM-Ⅲ-R | 60+ | 12628 | 80 | 8 |
| Wei et al., 2008^[26]^ | Hebei | Both | Both | No | two | 100.00 | MMSE;  interview | CCMD-Ⅱ-R | 60+ | 2308 | 113 | 8.5 |
| Kang et al., 2011^[27]^ | Hebei | Both | Both | Yes | two | 95.60 | MMSE;  ADL;  interview | DSM-IV;  NINCDS-ADRDA | 60+ | 3632 | 177 | 10 |
| Li et al., 2008^[28]^ | Hebei | Both | Both | No | two | 98.52 | MMSE;  BSSD | DSM-Ⅳ | 65+ | 2126 | 166 | 9.5 |
| Yu et al., 2011^[29]^ | Hebei | Mixed | NA | No | two | 96.02 | MMSE;  MoCA;  ADL; | DSM-IV;  NINCDS-ADRDA | 80+ | 1086 | 86 | 9 |
| Yu et al., 2004^[30]^ | Hebei | Mixed | NA | Yes | two | 96.60 | MMSE;  ADL;  HIS | DSM-IV;  NINCDS-ADRDA | 65+ | 2674 | 88 | 9.5 |
| Wu et al., 2002^[31]^ | Henan | Both | Rural | Yes | two | 73.39 | MMSE;  ADL;  HIS | DSM-IV | 55+ | 14335 | 299 | 8 |
| Sun et al., 2012^[32]^ | Heilongjiang | Both | Both | Yes | two | 92.82 | MMSE;  ADL;  HIS | DSM-Ⅳ;  NINCDS-ADRDA | 60+ | 3698 | 87 | 11 |
| Tan et al., 2007^[33]^ | Hubei | Both | Mixed | No | two | 100.00 | MMSE;  ADL;  HIS | DSM-Ⅲ-R;  NINCDS-ADRDA | 55+ | 3908 | 78 | 10 |
| Yang et al., 2002^[34]^ | Hunan | Both | Urban | Yes | two | 85.98 | MMSE;  ADL;  HIS | DSM-Ⅲ-R | 60+ | 2257 | 34 | 9.5 |
| Fan et al., 2000^[35]^ | Jiangsu | Mixed | Mixed | No | two | 100.00 | MMSE;  HDS | DSM-Ⅲ-R;  NINCDS-ADRDA | 60+ | 3268 | 31 | 9 |
| Wu et al., 2017^[36]^ | Jiangsu | Both | Both | No | two | 93.02 | MMSE;  ADL;  HIS | NINCDS-ADRDA | 60+ | 4195 | 205 | 10 |
| Liu et al., 2008^[37]^ | Jiangxi | Mixed | NA | No | two | 93.87 | MMSE;  HIS | ICD-10;  CCMD-Ⅲ-R | 60+ | 1408 | 77 | 8 |
| Lu et al., 2007^[38]^ | Jiangxi | Both | NA | No | two | 92.05 | MMSE;  HIS;  interview | ICD-10;  CCMD-Ⅲ | 60+ | 2408 | 113 | 8.5 |
| Yuan et al., 2005^[39]^ | Jiangxi | Both | NA | No | two | 100.00 | ADL;  HIS | ICD-10;  CCMD-Ⅲ | 60+ | 2126 | 72 | 7.5 |
| Liao et al., 2015^[40]^ | Jiangxi | Both | Urban | Yes | two | 100.00 | MMSE;  interview | ICD-10;  NINCDS-ADRDA | 60+ | 9733 | 432 | 8 |
| Wu et al., 2012^[41]^ | Jiangxi | Both | NA | No | two | 90.44 | MMSE;  ADL | ICD-10;  CCMD-Ⅲ | 60+ | 4350 | 214 | 10 |
| He et al., 2012^[42]^ | Jiangxi | Both | Rural | Yes | two | 86.80 | CSI-D;  GMSA;  interview | CCMD-Ⅲ | 65+ | 1029 | 29 | 8 |
| Sun et al., 2001^[43]^ | Liaoning | Mixed | NA | No | two | 100.00 | MMSE;  HDS | DSM-Ⅳ | 60+ | 2000 | 48 | 7.5 |
| Wang et al., 2010^[44]^ | Liaoning | Both | NA | Yes | two | 97.04 | HDS;  CMS | DSM-Ⅳ-R;  NINCDS-ADRDA | 60+ | 2100 | 76 | 8.5 |
| Wu et al., 2011^[45]^ | Liaoning | Mixed | NA | No | one | 100.00 | interview | DSM-IV | 60+ | 1050 | 72 | 6 |
| HuRiLe et al., 2013^[46]^ | Inner Mongolia | Both | Urban | No | two | 92.34 | MMSE;  HIS | DSM-Ⅳ;  NINCDS-ADRDA | 55+ | 9266 | 448 | 9 |
| Li et al., 2003^[47]^ | Inner Mongolia | Mixed | Urban | No | two | 100.00 | MMSE;  ADL;  HIS | ICD-10 | 60+ | 2324 | 31 | 8.5 |
| Shang et al., 2015^[48]^ | Qinghai | Both | Rural | Yes | two | 88.37 | MMSE;  ADL;  interview | DSM-Ⅳ;  NINCDS-ADRDA | 60+ | 3974 | 53 | 9 |
| Zhu et al., 2002^[49]^ | Shandong | Both | Urban | No | one | 94.30 | MMSE | DSM-IV-R;  NINCDS -ADRDA | 60+ | 2014 | 57 | 7.5 |
| Shao et al., 2016^[50]^ | Shandong | Both | Rural | No | two | 79.08 | MMSE;  ADL;  HIS | NIA-AA criteria | 65+ | 2377 | 127 | 8.5 |
| Cui et al., 2013^[51]^ | Shandong | Mixed | NA | Yes | two | 80.00 | MMSE;  MoCA;  ADL | DSM-IV;  NINCD-ADRDA | 60+ | 601 | 21 | 8 |
| Wang et al., 2010^[52]^ | Shandong | Both | Urban | No | two | 100.00 | MMSE;  ADL | ICD-10;  DSM-IV | 60+ | 1500 | 77 | 8.5 |
| Zhang et al., 2016^[53]^ | Shandong | Both | NA | Yes | two | 100.00 | MMSE;  ADL | CCMD-Ⅱ-R | 65+ | 7964 | 413 | 9 |
| Wang et al., 2009^[54]^ | Shandong | Both | NA | Yes | two | 96.71 | MMSE | DSM-IV;  NINCDS-ADRDA | 60+ | 618 | 42 | 7 |
| Fan et al., 2011^[55]^ | Shanxi | Mixed | NA | Yes | two | 100.00 | MMSE;  ADL | DSM-IV;  NINCDS-ADRDA | 60+ | 1826 | 38 | 8.5 |
| Wang et al., 2011^[56]^ | Shanxi | Both | NA | Yes | two | 100.00 | interview | NINCDS-ADRDA | 65+ | 5964 | 290 | 8 |
| Qu et al.,2004^[57]^ | Shaanxi | Both | Both | Yes | two | 94.80 | MMSE;  ADL;  HIS | DSM-IV;  NINCDS-ADRDA | 55+ | 4850 | 100 | 10 |
| Zhou et al., 2002^[58]^ | Shanghai | Both | Both | Yes | three | 94.20 | MMSE;  ADL;  HIS | DSM-Ⅳ;  NINCDS-ADRDA | 55+ | 15910 | 344 | 11 |
| Zhu et al., 1998^[59]^ | Shanghai | Both | Urban | Yes | two | 100.00 | MMSE | DSM-Ⅲ-R;  NINCDS-ADRDA | 60+ | 3083 | 110 | 8 |
| Zhang et al., 2001^[60]^ | Shanghai | Mixed | Urban | Yes | two | 87.66 | MMSE;  ADL;  HIS | ICD-10 | 55+ | 1186 | 9 | 8 |
| Ding et al., 2013^[61]^ | Shanghai | Mixed | Urban | No | two | 69.50 | MMSE;  ADL;  CES-D | DSM-IV; NINCDS-ADRDA | 60+ | 3141 | 113 | 8 |
| Fei et al., 2001^[62]^ | Shanghai | Both | NA | Yes | two | 91.30 | MMSE | DSM-Ⅲ-R | 55+ | 462 | 7 | 6.5 |
| Wang et al., 1995^[63]^ | Shanghai | Both | Rural | Yes | two | 83.38 | MMSE;  ADL | DSM-Ⅲ-R;  NINCDS-ADRDA | 55+ | 1515 | 27 | 10.5 |
| Lou et al., 1998^[64]^ | Shanghai | Mixed | Rural | Yes | two | 88.20 | MMSE;  HIS | DSM-Ⅲ-R; | 50+ | 2316 | 23 | 8.5 |
| Sun et al., 2012^[65]^ | Shanghai | Both | Urban | Yes | two | 71.70 | MMSE;  CSDD;  ADL | NIA-AA criteria;  DSM-IV-R | 60+ | 1472 | 56 | 7 |
| Tang et al., 2003^[66]^ | Sichuan | Both | NA | Yes | two | 77.81 | MMSE;  ADL;  HIS | DSM-Ⅲ-R;  NINDS-ADRDA | 55+ | 2847 | 25 | 8.5 |
| Tang et al., 2005^[67]^ | Sichuan | Both | Both | No | two | 93.83 | MMSE;  ADL;  HIS | DSM-Ⅲ-R;  NINDS-ADRDA | 55+ | 9261 | 187 | 10 |
| Tang et al., 1999^[68]^ | Sichuan | Both | Rural | No | two | 100.00 | MMSE;  HDS | DSM-Ⅲ-R;  NINDS-ADRDA | 65+ | 5987 | 86 | 10 |
| Li et al., 2015^[69]^ | Tianjin | Both | NA | Yes | two | 100.00 | MMSE;  ADL | DSM-IV;  NINDS-ADRDA | 60+ | 2532 | 144 | 8.5 |
| Meng et al., 2014^[70]^ | Sinkiang | Both | Mixed | Yes | two | 98.55 | MMSE;  ADL;  HIS | DSM-IV;  NINCDS-ADRD | 55+ | 3610 | 197 | 10 |
| Zhou et al., 2008^[71]^ | Sinkiang | Both | Mixed | Yes | two | 100.00 | MMSE;  ADL;  HIS | DSM-IV; NINCDS-ADRDA | 50+ | 8284 | 303 | 10 |
| Sun et al., 2002^[72]^ | Yunnan | Both | Both | No | one | 100.00 | MMSE | MMSE | 60+ | 6476 | 81 | 6 |
| Liu et al., 2017^[73]^ | Yunnan | Mixed | NA | Yes | two | 97.91 | MMSE;  interview | NINCDS-ADRDA | 60+ | 3977 | 93 | 10 |
| Zhou et al., 2014^[74]^ | Zhejiang | Mixed | NA | No | two | 100.00 | MMSE;  ADL | ICD-10 | 60+ | 121949 | 4795 | 9 |
| Zou et al., 2002^[75]^ | Chongqing | Mixed | Urban | No | two | 87.35 | MMSE;  HIS;  interview | DSM-IV-R | 65+ | 1519 | 73 | 8.5 |

AD, Alzheimer’s disease; ADL, Activity of Daily Living Scale; BSSD, Brief screening scale for dementia; CCMD-Ⅱ-R/-Ⅲ/-Ⅲ-R, Chinese Classification of Mental Disorders Version 2, Revised/ Version 3/Version 3, Revised; CDR, Clinical Dementia Rating Scale; CES-D, Center for epidemiological survey-Depression Scale; CSDD, Cornell Scale for Depression in Dementia; CSI-D, Community Screening Instrument for Dementia; DSM-Ⅲ-R/-IV/-IV-R, Diagnostic and Statistical Manual of Mental Disorder Third Edition, Revised/Fourth Edition/Fourth Edition, Revised; GMSA, Geriatric Mental State Schedule Shortened Community Version; HDS, Hasegawa’s Dementia Scale; HIS, Hachinski Ischemic Score; ICD-10, International Classification of Diseases 10th editions; Mixed, not discriminated; MMSE, Mini-Mental State Examination; MoCA, Montreal Cognitive Assessment Scale; NA, not available; NIA-AA criteria, National Institute on Aging and Alzheimer's Association criteria; NINCDS-ADRDA, National Institute of Neurological and Communicative Diseases and Stroke/Alzheimer's Disease and Related Disorders Association; No, not provided; Yes, provided.

**Table S2.** Detailed characteristics of 24 PD studies

| Reference | Location | Gender | Age  range | Sample  size | Cases | Phase design | Response rate (%) | Screening | Diagnosis | Quality score |
| --- | --- | --- | --- | --- | --- | --- | --- | --- | --- | --- |
| Liu et al., 2010^[76]^ | Sinkiang | Mixed | 55+ | 6145 | 57 | two | 84.72 | UPDRS;  interview | ≥3 of 5 cardinal signs | 10 |
| Wang et al., 2013^[77]^ | Sinkiang | Both | 35+ | 9732 | 141 | two | 96.53 | interview | UK-PDSBBC | 8 |
| Zhang et al., 2013^[78]^ | Sinkiang | Both | 35+ | 5113 | 62 | two | 100.00 | UPDRS;  interview | UK-PDSBBC | 10 |
| Song et al., 2012^[79]^ | Sinkiang | Both | 45+ | 5932 | 88 | three | 100.00 | interview | ≥1 Insidiously progressive rest tremor, rigidity, hypokinesia + no definite cause+ middle age and levodopa responsiveness | 10 |
| Wang et al., 2010^[80]^ | Beijing | Mixed | 60+ | 3473 | 70 | two | 99.19 | MMSE;  ADL;  GDS | UK-PDSBBC | 8 |
| Shang et al., 2017^[81]^ | Beijing | Both | 40+ | 2326 | 44 | two | 95.70 | screening questionnaire | The 2015 International Association  of dyskinesia (MDS) | 7.5 |
| Zhu et al., 2014^[82]^ | Beijing | Mixed | 65+ | 1188 | 62 | One | 100.00 | CDR;  MMSE | UK-PDSBBC | 7 |
| Yu et al., 2007^[83]^ | Hebei | Both | 65+ | 2674 | 50 | One | 96.60 | screening questionnaire; interview | ≥2 cardinal signs and exclusion of other causes | 7.5 |
| Yang et al., 2017^[84]^ | Fujian | Both | 65+ | 1771 | 32 | one | 99.49 | UPDRS;  interview | Chinese diagnostic criteria for Parkinson's disease. | 8.5 |
| Qiao et al., 2001^[85]^ | Shaanxi | Both | 55+ | 4800 | 38 | two | 94.76 | interview | ≥2 cardinal signs, and ≥1 static tremor and action reduction and  exclusion of other causes | 9 |
| Gao et al., 2016^[86]^ | Shaanxi | Both | 0+ | 41538 | 24 | two | 100.00 | interview | ≥2 cardinal signs, and ≥1 static tremor and action reduction and  exclusion of other causes | 8 |
| Liu et al.,2002^[87]^ | Henan | Both | 60+ | 16488 | 86 | one | 73.00 | screening questionnaire;  physical examination | UK-PDSBBC | 7 |
| Qin et al., 2000^[88]^ | Sichuan | Both | 0+ | 36835 | 20 | two | 100.00 | interview | ≥2 cardinal signs, and ≥1 static tremor and action reduction and exclusion of other causes | 8 |
| Zhou et al., 2001^[89]^ | Shanghai | Both | 55+ | 16030 | 158 | two | 94.20 | screening questionnaire;  interview;  physical examination | resting tremor, bradykinesia, or rigidity, in the absence of other apparent causes of parkinsonism | 9 |
| Zou et al., 2014^[90]^ | National | Both | 60+ | 9396 | 225 | two | 83.46 | interview;  UPDRS | UK-PDSBBC | 9 |
| Ho et al., 1989^[91]^ | Hongkong | Both | 60+ | 561 | 19 | one | 73.60 | Interview;  physical examination | ≥3 of 5 cardinal signs, or ≥2 cardinal signs if there is ≥ 2 additional condition | 6 |
| Woo et al., 2004^[92]^ | Hongkong | Both | 55+ | 415 | 2 | one | 73.50 | screening questionnaire; interview;  physical examination | resting tremor, bradykinesia, or rigidity, in the absence of other apparent causes of parkinsonism | 5.5 |
| Wang et al., 1994^[93]^ | Taiwan | Mixed | 50+ | 482 | 6 | two | 70.60 | interview;  UPDRS | ≥ 2 of 4 cardinal signs if not receiving antiparkinsonian drugs;  or, ≥ 1 of 4 cardinal signs if improved by medications | 7.5 |
| Chen et al., 2001^[94]^ | Taiwan | Both | 40+ | 10058 | 37 | two | 88.10 | screening questionnaire; interview | ≥2 cardinal signs and exclusion of other causes | 7 |
| Chen et al., 2009^[95]^ | Taiwan | Both | 40+ | 11332 | 80 | two | 84.90 | interview;  UPDRS;  physical examination | ≥2 cardinal signs and exclusion of other causes | 9 |
| Wang et al., 1996^[96]^ | Taiwan | Both | 50+ | 3915 | 23 | one | 96.00 | screening questionnaire; UPDRS;  physical examination | ≥2 of 4 cardinal signs if not receiving antiparkinsonian drugs; or, ≥1 of 4 cardinal signs if improved by medications | 9 |
| Wang et al., 2018^[97]^ | Guangxi | Both | 40+ | 3111 | 47 | two | 100.00 | screening questionnaire; interview | ≥3 of 5 cardinal signs and exclusion of other causes | 9 |
| Li et al., 1985^[98]^ | Changsha, Chengdu, Guangzhou, Harbin, Shanghai, Yinchuan | Mixed | 50+ | 14186 | 28 | two | 100.00 | interview;  physical examination | resting tremor, bradykinesia, or rigidity, in the absence of other apparent causes of parkinsonism | 9 |
| Zhang et al., 2005^[99]^ | Beijing, Xian, Shanghai | Both | 55+ | 29454 | 283 | three | 94.00 | interview;  physical examination | ≥3 cardinal signs, or ≥2 cardinal signs if there is ≥1 additional condition: asymmetry, one sign was resting tremor or bradykinesia, or no levodopa unresponsiveness | 9 |

ADL, Activity of Daily Living Scale; CDR, Clinical Dementia Rating Scale; GDS, Geriatric Depression Scale; Mixed, not discriminated; MMSE, Mini-Mental State Examination; NA, not available; PD, Parkinson’s disease; UK-PDSBBCUK, Parkinson’s disease Society Brain Bank Criteria; UPDRS, Unified Parkinson's Disease Rating Scale;

**Table S3.** Prevalence of AD and PD in subgroups with different diagnostic criteria.

|  | Diagnostic criteria | Studies, n | Cases, n | Population, n | Prevalence (95% CI), % | I^2^, % | *P* |
| --- | --- | --- | --- | --- | --- | --- | --- |
| **AD** | DSM-Ⅲ-R | 5 | 185 | 26551 | 1.01 (0.43-2.39) | 96.9 | <0.01 |
|  | DSM-IV(-IV-R) | 5 | 552 | 18217 | 3.23 (2.05-5.08) | 96.0 | <0.01 |
|  | DSM-Ⅲ-R;  NINCDS-ADRDA | 6 | 376 | 21370 | 1.59 (1.01-2.50) | 94.6 | <0.01 |
|  | DSM-IV(-IV-R);  NINCDS-ADRDA | 23 | 2762 | 77604 | 3.48 (2.83-4.19) | 96.2 | <0.01 |
|  | Mixed | 7 | 1041 | 22997 | 4.51 (4.06-4.99) | 59.5 | 0.02 |
|  | Other-A | 7 | 5375 | 142985 | 2.95 (1.94-4.16) | 97.9 | <0.01 |
| **PD** | UK-PDSBBC | 3 | 374 | 23520 | 1.71 (0.51-2.90) | 98.0 | <0.01 |
|  | Other-P | 11 | 462 | 50446 | 0.99 (0.60-1.62) | 96.3 | <0.01 |

CCMD-Ⅱ-R/-Ⅲ/-Ⅲ-R, Chinese Classification of Mental Disorders Version 2, Revised/ Version 3/Version 3, Revised; DSM-Ⅲ-R/-IV/-IV-R, Diagnostic and Statistical Manual of Mental Disorder Third Edition, Revised/Fourth Edition/Fourth Edition, Revised; ICD-10, International Classification of Diseases 10th editions; Mixed, multiple diagnostic criteria including ICD-10, CCMD-Ⅲ/-Ⅲ-R, DSM-IV/-IV-R, NINCDS-ADRDA and NIA-AA criteria; MMSE, Mini-Mental State Examination; NIA-AA criteria, National Institute on Aging and Alzheimer's Association criteria; NINCDS-ADRDA, National Institute of Neurological and Communicative Diseases and Stroke/Alzheimer's Disease and Related Disorders Association; Other-A, ICD-10, NINCDS-ADRDA, CCMD-2-R and MMSE; Other-P, diagnostic criteria other than the UK-PDSBBC; UK-PDSBBC, UK Parkinson’s disease Society Brain Bank Criteria.

**Table S4.** The seven geographic regions in China.

| Region | Included provinces |
| --- | --- |
| North China | Beijing Municipality, Hebei province, Inner Mongolia Autonomous Region, Shanxi province, Tianjin Municipality |
| Northeast China | Heilongjiang province, Jilin province, Liaoning province |
| East China | Anhui province, Fujian province, Jiangsu province, Jiangxi province, Shandong province, Shanghai Municipality, Zhejiang province, Taiwan district |
| South China | Guangdong province, Guangxi Zhuang Autonomous Region, Hainan province, Hong Kong Special Administrative Region, Macao Special Administrative Region |
| Central China | Henan province, Hubei province, Hunan province |
| Southwest China | Chongqing Municipality, Guizhou province, Sichuan province, Tibet Autonomous Region, Yunnan province |
| Northwest China | Gansu province, Ningxia Hui Autonomous Region, Qinghai province, Shaanxi province, Sinkiang Uyghur Autonomous Region |

**Reference**

1. Wang, T.X., Sun, J.Z., Wei, X.B., Xi, X.Y., Wu, Q.Y., Lian, X.L., et al. (1999). Epidemiology of dementia in Urban and Rural areas of Anhui Province (in Chinese). *Occupation and Health* 15(9), 43-45.
2. Li, L., Li, F., Ma, Y., Chen, R., Liu, Z.M., Qin, X., et al. (2011). Prevalence of dementia among the elderly in rural community of Anhui (in Chinese). *Chinese Journal of Disease Control & Prevention* 15(04), 292-294.
3. Xing, W.C. (2018). Epidemiology, preventive intervention strategies of senile dementia in a certain area (in Chinese). *Special Health* 13, 1-2.
4. Tang, Z., Meng, C., Dong, H.Q., Wu, X.G., Min, B.Q., Zhang, X., et al. (2002). The prevalence of senile dementia in Beijing (in Chinese). *Chinese Journal of Gerontology* 22(04), 244-246.
5. Xie, H.G., Wang, L.N., Wang, X.H., Wang, Z.F., and Qi, Z.F. (2000). Dementia in male veterans: a preliminary study of its prevalence and risk factors (in Chinese). *Medical Journal of Chinese People's Liberation Army* 25(05), 365-367.
6. Cui, B. (2012). A case-control study on the prevalence and risk factors of Alzheimer's disease in retired military cadres stationed in Beijing. [master’s thesis]. [Tianjin]: Nankai University
7. Li, Z.J., Tong, Z.F., Jiang, Z.Y, Diao, G.R., Lv, X.Z., and Shi, Q.K. (1999). Investigation on the prevalence of dementia among the elderly in Jianguomen Street, Dongcheng District, Beijing (in Chinese). *Chinese Journal of Gerontology* 19(01), 3-5.
8. Zhang, J.L., Zhang, H.H., Tao, G.S., Liu, X.L., Wu, Q., Ji, X.L., et al. (1998). An Epidemiological Study on Senile Dementia among 1390 Elderly People in Haidian District, Beijing (in Chinese). *Chinese Journal of Epidemiology* 19(01), 3-5.
9. Zheng, X.X. (2010). An epidemiologic survey of Alzheimer’s disease in Mentougou District of Beijing (in Chinese). *Journal of Neuroscience and Mental Health* 10(2), 196-198.
10. Chen, B., Luo, W.W., Chen, L.L., Shi, G.Q., Chen, Y.P., Li, L.E., et al. (2009). Condition of senile dementia and analysis of its dangerous social-psychological factors (in Chinese). *Fujian Medical Journal* 31(01), 133-136.
11. Li, H., Zhang, H.H., Huang, H., Wang, Y.Z., and Huang, H.L. (2009). Prevalence of dementia among rural elderly in Gushan township, Fuzhou (in Chinese). *Chinese Journal of Epidemiology* 30(08), 772-775.
12. Zhang, Q.Y., Wan, X.H., Zhang, M., Sun, X., and Zhang, X.T. (2017). Epidemiological investigation and related factors of Alzheimer's disease in Lanzhou (in Chinese). *Journal of Imaging Research and Medical Applications* 1(10), 222-224.
13. Wang, G.P., Pei, G.X., Xie, R., Ding, Z.J., Zhang, Y.L., Du, H.N., et al. (2016). Epidemiological status investigation of senile dementia in the people aged 65 and older of Tianshui city (in Chinese). *Medical Journal of Chinese People’s Health* 28(15), 52-55.
14. Huang, H.M., Chen, S., Zhao, Y.J., Zou, L.Y., and Zhang, R. (2014). Analysis on the prevalence and influencing factors of Alzheimer's disease among the elderly in Shenzhen community (in Chinese). *Chinese Journal of Practical Nervous Diseases* 17(13), 50-52.
15. Liang, L.J., Lin, Q.M., Huang, C., Lin, Z.Y., Zheng, W.Z., and Zhang, W.F. (2003). Epidemiological survey of senile dementia among elder people in community (in Chinese). *Modern Preventive Medicine* 30(4), 515-516.
16. Xue, G.H., Shao, Y.C., Zhu, G.Z., Shi, M.H., Zhou, L.X., Zhou, X.P., et al. (1997). Epidemiology of dementia in Guangdong Province (in Chinese). *The Journal of Practical Medicine* 13(6), 371-372.
17. Tang, M.N., Ma, C., Huang, X.M., Han, H.Y., Guo, Y.B., Huang, J.M., et al. (2007). The prevalence of dementia in urban and rural areas in Guangzhou (in Chinese). *Chinese Journal of Nervous and Mental Diseases* 33(06), 340-344.
18. Ma, C., Tang, M.N., Guo, Y.B., Han, H.Y., Huang, X.M., Huang, J.M., et al. (2005). The prevalence of dementia in the urban and rural aged in Guangzhou (in Chinese). *Chinese Journal of Psychiatry* 38(04), 39-42.
19. Lai, S.L., Wen, Z.H., Liang, W.X., Xie, H., Wang, Q., Elena, Y., et al. (2000). Prevalence of dementia in an urban population aged≥75 years in Guangzhou (in Chinese). *Chinese Journal of Geriatrics* 19(6), 450-455.20.
20. Gao, Q.W., Huang, C.Y., Liu, J., and Yang, M.C. (2004). Investigation on the prevalence of dementia in retired military cadres (in Chinese). *Guangdong Medical Journal* 25(2), 203-204.
21. Yu, J.L., Feng, R.M., Fang, S.X., Lu, Y., Zhou, W.C., and Jiang M.J. (1998). Investigation on the prevalence of senile dementia in rural areas in coastal areas of Guangdong Province (in Chinese). *Chinese Journal of Nervous and Mental Diseases* 24(01), 3-5.
22. Li, H.H., Hu, C.Y., Wei, D.M., Fu, M., Lv, Z.P., Deng, M.Y., et al. (2015). Prevalence of dementia among elderly people in Santang community, Nanning city (in Chinese). *Chinese Journal of Geriatric Care* 13(1), 29-31.
23. Huang, W.Y., Yang, X., Yang, J.Y., and Deng, H.C. (2007). Investigation on prevalence of dementia among elderly in urban communities of Guiyang city (in Chinese). *Chinese Journal of Public Health* 23(8), 983-985.
24. Wu, C.D., Huang, J.D., Chen, W.W., Chen, S.S., Gong, J.B., Kuang, B.X., et al. (2003). Epidemiological investigation of patients with Alzheimer' s dementia in Urban area in Hainan Province (in Chinese). *China Tropical Medicine* 3(5), 690-691.
25. Chen, W.W., Wu, C.D., Huang, J.D., Chen, S.S., Gong, J.B., Kuang, B.X., et al. (2004). Epidemiological survey of Alzheimer's Disease and cerebrovascular dementia in Hainan (in Chinese). *China Tropical Medicine* 4(6), 1056-1076.
26. Wei, H.M., Zhang, H.J., and Xu, L. (2008). Epidemiological investigation of senile dementia in Baoding (in Chinese). *Modern Preventive Medicine* 35(5), 847-848.
27. Kang, M.Y., Gao, Y.M., Huo, H.Q., Chen, Y.M., Wang, J., Li, M.J., et al. (2011). Epidemiological features of chronic and Alzheimer’s diseases in the community-based elderly living in cities and countries in Hebei province (in Chinese). *Chinese Journal of Epidemiology* 32(7), 672-675.
28. Li, K.Q., Jiang, Q.P., Cui, L.J., Cui, Z., Li, J.F., Yang, L.H., et al. (2008). Epidemiological Investigation of Alzheimer Disease in the Urban and Rural Areas in Hebei Province (in Chinese). *China Journal of Health Psychology* 16(11), 1251-1253.
29. Yu, B.C., Xu, R.H., Wei, W.Z., Zhong, W.H., He, J.Z., Qi, C.W., et al. (2011). Study of the prevalence of dementia and major subtypes in senile male veterans (in Chinese). *People’s Military Surgeon* 54(S1), 36-38.
30. Yu, B.C., Ouyang, L.S., Pan, Z.G., Wei, S.X., Wang, Y.M., and Wang, C.Z. (2004). A Survey on the Prevalence of Dementia and Major Subtypes in Elderly Veteran (in Chinese). *Chinese General Practice* 7(19), 1404-1406.
31. Wu, C.S., Zhou, D.F., Peter, C., Zhang, L., Karl, K., Fan, J.H., et al. (2002). Investigation on the prevalence of Alzheimer's disease in Lin County, Henan Province (in Chinese). *Chinese Journal of Psychiatry* 35(4), 65-66.
32. Sun, Z.H., Cui, G.C., Feng, H.L., and Han, Y.F. (2011). Research on Prevalence of Alzheimer’s Disease of Fulaerji District in Hei Long Jiang Province (in Chinese). *China Journal of Health Psychology* 19(03), 278-280.
33. Tan, J.H. (2007). Investigation and Analysis on the prevalence of senile dementia in ethnic areas of Hubei Province (in Chinese). *The Medical Forum* 11(8),703-705.
34. Yang, Q.D., Zhou, Y.H., Tan, X.L., Du, X.P., Xia, J., Zhang, L., et al. (2002). Study of senile dementia epidemiology of old-aged population in urban area with high incidence of cerebrovascular disease (in Chinese). *Chinese Journal of Clinical Rehabilitation* 6(01), 28-29.
35. Fan, J.X., Yan, J.L., Chen, Z.H., Liu, J., Zhang, X.R., Duanmu, X.R., et al. (2000). An epidemiological report of senile dementia in Nanjing area (in Chinese). *Journal of Clinical Psychiatry* 10(3),137-138.
36. Wu, Y., Cheng, Z.H., Bao, Z.H., Fan, J., Tang, L., Guo, T., et al. (2017). Survey on the status of Alzheimer’s disease and psychological health of family caregivers in Wuxi (in Chinese). *Chinese Preventive Medicine* 18(11), 840-846.
37. Liu, Q.Q., Zhao, X.H., and Huang, H.L. (2008). Investigation on the distribution of nine chemical elements among females Alzheimer's Disease (AD) patients' blood and relationship between different levels of elements and pathogenesis of AD (in Chinese). *Chinese Journal of Disease Control & Prevention* 12(06), 587-590.
38. Lu, Q.B. (2007). Study on the epidemic situation of Alzheimer's Disease and the Distribution characteristics of Chemical elements in whole Blood of patients in Hongdu Community of Nanchang City. [master’s thesis]. [Nanchang]: Nanchang University
39. Yuan, Y.F., Wan, A.L., Chen, J.Y., Chao, X.L., Huang, G.M., Zhou, X.J., et al. (2005). Epidemiological report of senile dementia in Nanchang city (in Chinese). *Chinese Journal of Neuromedicine* 4(01), 65-67.
40. Liao, J., Huang, H.L., Yan, J., Ma, J., Tao, X.Q., Liao, X., et al. (2015). The prevalence of Alzheimer’s disease in Nanchang community and its influencing factors (in Chinese). *Chinese Journal of Gerontology* 35(24), 7176-7177.
41. Wu, L., Lai, D., Tang, J., Yuan, Y.F., and Huang, H.L. (2012). Evaluation on Quality of Life among Alzheimer’s Disease Patients in Urban Community (in Chinese). *Journal of Chengdu Medical college* 7(03), 367-370.
42. He, X.S. (2012). Analysis on the prevalence and influencing factors of senile dementia in rural community (in Chinese). *Journal of jiujiang University (natural sciences)* 27(01), 29-32.
43. Sun, Y.J., Xun, M.M., Wu, C.H., Yu, H., Tang, Y.N., Mu, L.B., et al. (2001). A sampling survey on the prevalence of senile dementia in Dalian (in Chinese). *Chinese Journal of Psychiatry* 34(01), 26.
44. Wang, Y., Jiang, C.L., Gao, Y., Xing, G.L., Yang, F.W., and He, S.Y. (2010). Epidemiological investigation of senile dementia in military dormitory in Dalian area (in Chinese). *Chinese Journal of Convalescent Medicine* 19(06), 567-569.
45. Wu, M., Liu, W.P., Li, Y.F., Chen, M., and Gao, F. (2011). Incidence status and comprehensive recuperation scheme of Alzheimer's disease in elderly convalescent patients (in Chinese). *Chinese Journal of Gerontology* 31(23), 4659-4660.
46. Huriletemuer, Zhang, C.Y., Zhao, S.G., Niu, G.M., A, R., Wang, Z. G., et al. (2013). Study of Alzheimer disease prevalence of Mongolian and Han populations in Inner Mongolia pasturing area (in Chinese). *Journal of Clinical Neurology* 26(01), 1-4.
47. Li, W.B., Wang, Y., Wei, L.Q., Shi, R.L., and Xu, C.Y. (2003). Investigation on the prevalence of senile dementia among some retired workers in Baotou City (in Chinese). *Literature and Information On Preventive Medicine* 9(6), 643-644.
48. Shang, Y. (2015). The risk factors of Alzheimer’s disease among Tibetan aged 60 years and older in Qinghai Province. [master’s thesis]. [Guangzhou]: Southern Medical University
49. Zhu, J.Y., Xu, W.L., Gao, Y., Xu, L.L., Yang, W.W., Shao, J.H., et al. An Epidemiological Study on Senile Dementia in Jinan Railway Area (in Chinese). *Journal of Modern Clinical Medicine* 28(01), 7-9.
50. Shao, W. (2016). The investigation and analysis of the gender differences in Alzheimer in rural areas. [master’s thesis]. [Jinan]: Shandong University
51. Cui, L.Q., Fang, F., Wang, B.P., Wang, H.P., Cai, J.X., Zhao, L., et al. (2013). Investigation on the prevalence of dementia among retired cadres stationed in Qingdao (in Chinese). *Chinese Preventive Medicine* 14(05), 4-5.
52. Wang, F., Xu, P., Zhu, S.H., Zhang, S.L., and Tu, N. (2010). Survey on the awareness rate and prevalence of Alzheimer's disease in the urban area of Zaozhuang city (in Chinese). *Chinese Journal of Practical Nervous Diseases* 13(23), 9-11.
53. Zhang, C.H., Liu, H.F., and Zhu, B. (2016). Epidemiological survey study about Alzheimers disease among the community elderly in Zibo area (in Chinese). *Medical Laboratory Science and Clinics* 27(9), 21-23.
54. Wang, H.Y., Zhang, Y.Q., Chen, X.R., Xu, X.J., and Liu, Z.X. (2009). Epidemiological survey of senile dementia among elderly people in communities of Tai’an (in Chinese). *Chinese Journal of Public Health* 25(08), 899-900.
55. Fan, Q.H., and Zheng, J.Z. (2011). Investigation of prevalence of Alzheimer and risk factors (in Chinese). *Modern Preventive Medicine* 38(15), 3029-3030.
56. Wang, Y.P., Zhai, J.B., Zhu, F., Zhang, W.W., Yang, X.J., and Qu, C.Y. (2011). Prevalence of Alzheimer’s disease and its influencial factors among elderly people in communities (in Chinese). *Chinese Journal of Public Health* 27(07), 827-828.
57. Qu, Q.M., Qiao, J., Yang, J.B., Han, J.F., Luo, G.G., Zhang, H., et al. (2001). Study of the prevalence of senile dementia among elderly people in Xi'an, China (in Chinese). *Chinese Journal of Geriatrics* 20(04), 42-45.
58. Zhou, B., Hong, Z., Huang, M.S., Zeng, J., and Jin, M.H. (2001). Prevalence of dementia in Shanghai] urban and rural area (in Chinese). *Chinese Journal of Epidemiology* 22(05), 54-57.
59. Zhu, Z.Q., Chen, J.X., Zhang, M.Y., Yu, J.P., and Zhang, W.B. (1998). Five-year longitudinal study of the prevalence of dementia and Alzheimer's disease (in Chinese). *General Psychiatry* (S1), 6-8.
60. Wang, Z.X. (2000). Investigation on the distribution of Senile dementia patients in Baoshan District, Shanghai (in Chinese). *China Journal of Health Psychology*, 8(05), 536-538.
61. Ding, D., Zhao, Q.H., Guo, Q.H., Meng, H.J., Wang, B., Zhou, Y., et al. (2013). Prevalence Survey of Dementia among Elderly in a Urban Community in Shanghai (in Chinese). *Chinese Journal of Clinical Neurosciences* 21(01), 19-25.
62. Fei, W.M., Cheng, L., Wang, Y.M., Hu, X.R., Sun, C.J., and Gao, Z.X. (2001). An analysis on senile dementia prevalence at a community in Luwan district, Shanghai (in Chinese). *Sichuan Mental Health*, 14(01), 31-32.
63. Wang, D., and Bu, S.M. (1995). Investigation of senile dementia in Shanghai County (in Chinese). *General Psychiatry* 7(02), 76-78.
64. Lou, K.J., Zha, Y.L., Hu, D.J., Xu, Y.F., Yao, P.F., and Gao, Z.X. (1998). The prevalence survey of senile dementia of Qingpu County, Shanghai, China (in Chinese). *Shanghai Journal of Preventive Medicine* 10(04), 187-189.
65. Sun, H.X., Wu, Y.W., Ye, F.L., Zhou, Z.M., Wang, G., Jiang, G.X., et al. (2012). Prevalence of dementia among elderly people in Sheshan town, Songjiang district, Shanghai (in Chinese). *Journal of Internal Medicine Concepts & Practice* 7(02), 91-95.
66. Tang, M.N., Liu, X.H., Han, H.Y., Qiu, C.J., Wu, S., Lu, J., et al. (2003). Early signs of Alzheimer's disease in community population (in Chinese). *Chinese Journal of Psychiatry* 36(02), 15-19.
67. Tang, M.N., Liu, X.H., Lu, J., Qiu, C.J., Han, H.Y., Wu, S., et al. (2005). Comparison of prevalence of dementia between in 1997 and 2000 surveys in the urban and rural community in Chengdu (in Chinese). *Chinese Journal of Psychiatry* 38(03), 170-173.
68. Tang, M.N., Guo, Y.B., Xiang, M.Z., and Huang, M.S. (1999). Epidemiology of senile dementia and Alzheimer disease in the rural area (in Chinese). *Journal of Clinical Psychiatry* 9(01), 3-5.
69. Li, C.H., Yang, L.S., Zhao, L., Su, Z.C., Pan, P., Wang, Y.L., et al. (2015). Epidemiological investigation on the prevalence of senile dementia among the patients served by a community hospital in Beichen District, Tianjin City (in Chinese). *Practical Preventive Medicine* 22(03), 305-308.
70. Meng, X.L., Liu, T., Liu, Y.X., Ma, N., Shala, H., Yong, Y.X., et al. (2014). Survey of the incidence of elderly dementia of Kazak ethnic group in Xinjiang Uygur Autonomous Region (in Chinese). *Chinese Journal of Neurology* 47(07), 491-495.
71. Zhou, X.H., Hong, Y., Ma, L., Zhang, X.N., Miao, H.J., Gulizaer., et al. (2008). Xinjiang Uigur Autonomous Region Uigurs and Hans epidemiological survey of Alzheimer's disease and vascular dementia (in Chinese). *Chinese Journal of Neurology* 41(12), 797-801.
72. Sun, J.H., Hu, S.Y., Liu, F.Y., and Liu, J.T. (2002). A survey on senile dementia among 6476 elder people in Yunnan, China (in Chinese). *Chinese Journal of Preventive Medicine* 36(03), 40.
73. Liu, B.Y. (2017). Prevalence of Alzheimer's disease in parts of Yunnan Province. [master’s thesis]. [Kunming]: Kunming Medical University
74. Zhou, Z.P., Chen, L.S., Zhou, L., Shen, J.P., and Zhang, F.M. (2014). Survey on prevalence of Alzheimer’s disease in Haining city (in Chinese). *Chinese Journal of General Practice* 12(10), 1639-1641.
75. Zou, K.L., Qi, J., He, Y., Zhang, S.H., and Guan, L. (2002). A cross-sectional study of senile dementia in Liang Lukou street of yuzhong county of Chongqing (in Chinese). *Chinese Journal of Geriatrics* 21(06), 32-34.
76. Liu, Y., Zhang, X.Y., He, Y., Tang, Y.Z., Chen, R.H., Han, X.H., et al. (2010). Investigation on prevalence rate of Parkinson’s disease in population aged 55 years old and above in Kashi, Xinjiang between 2008 and 2009 (in Chinse). *Chinese Journal of Geriatrics* 43(12), 863-865.
77. Wang, L.N., Zhang, T.F., Wang, Y.L., and Yang, X.L. (2013). Study on the prevalence and relative factors of the Parkinson's Disease in residents aged 35 years or older in Urumqi city (in Chinse). *Journal of Xinjiang Medical University* 36(03), 278-281.
78. Zhang, J.L., Wang, Y.L., Yao, Y.N., and Yang, X.L. (2013). Analysis of prevalence and related factors in different national Parkinson's disease in Yili of Xinjiang area (in Chinse). *Journal of Xinjiang Medical University* 36(03), 273-277.
79. Song, H.X. (2012). Study on the Related Factors and Prevalence for the Parkinson's Disease of Uygur Residents in the area of Hetian, Xinjiang Uygur Autonomous Region. [master’s thesis]. [Xinjiang]: Xinjiang Medical University
80. Wang, L.N., Tan, J.P., Xie, H.G., Zhang, X., Wang, W., Wang, Z.F., et al. (2010). A cross-sectional study of neurological disease in the veterans of military communities in Beijing (in Chinse). *Chinese Journal of Internal Medicine* 49(06), 463-466.
81. Shang, M.Q., Wan, Z.R., Feng, T., and Du, J.C., (2017). Analysis of the Prevalence and Risk Factors for Parkinson' s Disease in middle-aged and Elderly Residents of Aerospace Center Hospital Adjacent Communities (in Chinse). *Neural Injury and Functional Reconstruction* 12(01), 28-31.
82. Zhu, M.W., Wu, W.P., Chen, S.H., Liu, M., Wang, L.N., He, Y., et al. (2014). The etiology of neurological disorders in 1188 elder patients with neuroimaging at geriatric outpatient clinic (in Chinese). *Chinese Journal of Internal Medicine* 53(03), 202-205.
83. Yu, B.C., He, J.Z., Cheng, P., Fang, H.Z., Wei, R., and Wang, Z. (2007). Prevalence of Parkinson Disease in elderly veterans (in Chinese). *China Healthcare Innovation* 2(19), 102-103.
84. Yang, H.Q., Liu, G.Q., Zhang, L.H., Xue, C., Lin, Y.Y., Lai, X.Q., et al. (2017). Study on the prevalence of Parkinson's disease and its relationship with metabolic syndrome among the elderly in Luoxing Street Mawei District (in Chinese). *Chinese Journal of Practical Nervous Disease* 20(24), 79-82.
85. Qiao, J., Qu, Q.M., Han, J.F., Yang, J.B., Luo, G.G., Zhang, H., et al. (2001). The Epidemiology of Parkinson Disease among Elderly People in Xi’an, China (in Chinese). *Chinese Journal of Neuroimmunology and Neurology* 8(02), 79-83.
86. Gao, X.R., Li, N., Wu, L.F., and Xue, Y.D. (2016). Status survey of Parkinson's disease in Northern Shaanxi, China (in Chinese). *Chinese Journal of Practical Nervous Diseases* 19(03), 82-83.
87. Liu, Y. (2010). The prevalence survey of Parkinson’s disease in the NIT in Linxian, China. [master’s thesis]. [Dalian]: Dalian Medical University
88. Qin, L.M., Wang, L.M. (2000). The prevalence survey of Parkinson's disease in Chengdu, China (in Chinese). *Guangxi Medical Journal* 22(05), 1169-1170.
89. Zhou, B., Hong, Z., Huang, M.S., Zeng, J., Jin, M.H., and Lv, C.Z. (2001). Prevalence of Parkinson's Disease in Shanghai urban and rural area (in Chinese). *Journal of Brain and Nervous Diseases* 9(06), 330-332.
90. Zou, Y.M. (2014). The epidemiological investigation of Parkinson’s disease in Chinese elderly veterans. [Doctoral thesis]. [Tianjin]: Nankai University
91. Ho, S.C., Woo, J., and Lee, C.M. (1989). Epidemiologic study of Parkinson’s disease in Hong Kong. *Neurology* 39(10), 1314-1318.
92. Woo, J., Lau, E., Ziea, E., and Chan, D.K.Y. (2004). Prevalence of Parkinson's disease in a Chinese population. *Acta Neurol Scand* 109(3), 228-231.
93. Wang, S.J., Fuh, J.L., Liu, C.Y., Lin, K.P., Chang, R., Yih, J.S., et al. (1994). Parkinson's Disease in Kin-Hu, kinmen: A Community Survey by Neurologists. *Neuroepidemiology*, 13, 69-74.
94. Chen, R. C., Chang, S. F., Su, C. L., Chen, T. H., Yen, M. F., Wu, H. M., et al. (2001). Prevalence, incidence, and mortality of PD: a door-to-door survey in Ilan county, Taiwan. *Neurology* 57(9), 1679-1686.
95. Chen, C.C., Chen, T.F., Hwang, Y.C., Wen, Y.R., Chiw, Y.H., Wu, C.Y., et al. (2009). Different Prevalence Rates of Parkinson’s Disease in Urban and Rural Areas: A Population-Based Study in Taiwan. *Neuroepidemiology*, 33(4), 350-357.
96. Wang, S.J., Fuh, J.L., Teng, E.L., Liu, C.Y., Lin, K.P., Chen, H.M., et al. (1996). A Door-to-Door Survey of Parkinson's Disease in a Chinese Population in Kinmen. *Archives of Neurology* 53(1):66-71.
97. Wang, S., Mo, Y.X., Peng, F., Wang, D.C., Luo, S.W., and Zheng, C.X. (2018). A study on the correlation between the prevalence of Parkinson's disease and dietary factors in Beihai, Guangxi, China (in Chinese). Chinese Journal of Geriatric Heart Brain and Vessel Diseases 20(4), 417-418.
98. Li, S.C., Schoenberg, B.S., Wang, C.C., Cheng, X.M., Rui, D.Y., Bolis, C.L., et al. (1985). A prevalence survey of Parkinson's disease and other movement disorders in the People's Republic of China. *Archives of Neurology* 42(7), 655-657.
99. Zhang, Z.X., Roman, G.C., Hong, Z., Wu, C.B., Qu, Q.M., Huang, J.B., et al. (2005). Parkinson’s disease in China: prevalence in Beijing, Xian, and Shanghai. *Lancet* 365(9459), 595-597.
