# Supplementary material for: Prevalence of Alzheimer's Disease and Parkinson's Disease in China: An Updated Systematical Analysis
Source: Front Aging Neurosci. 2020 Dec 21;12:603854. doi: 10.3389/fnagi.2020.603854 (PMC7793643; doi:10.3389/fnagi.2020.603854)
Supplement: Supplementary file 2 [file Image_1.PDF]

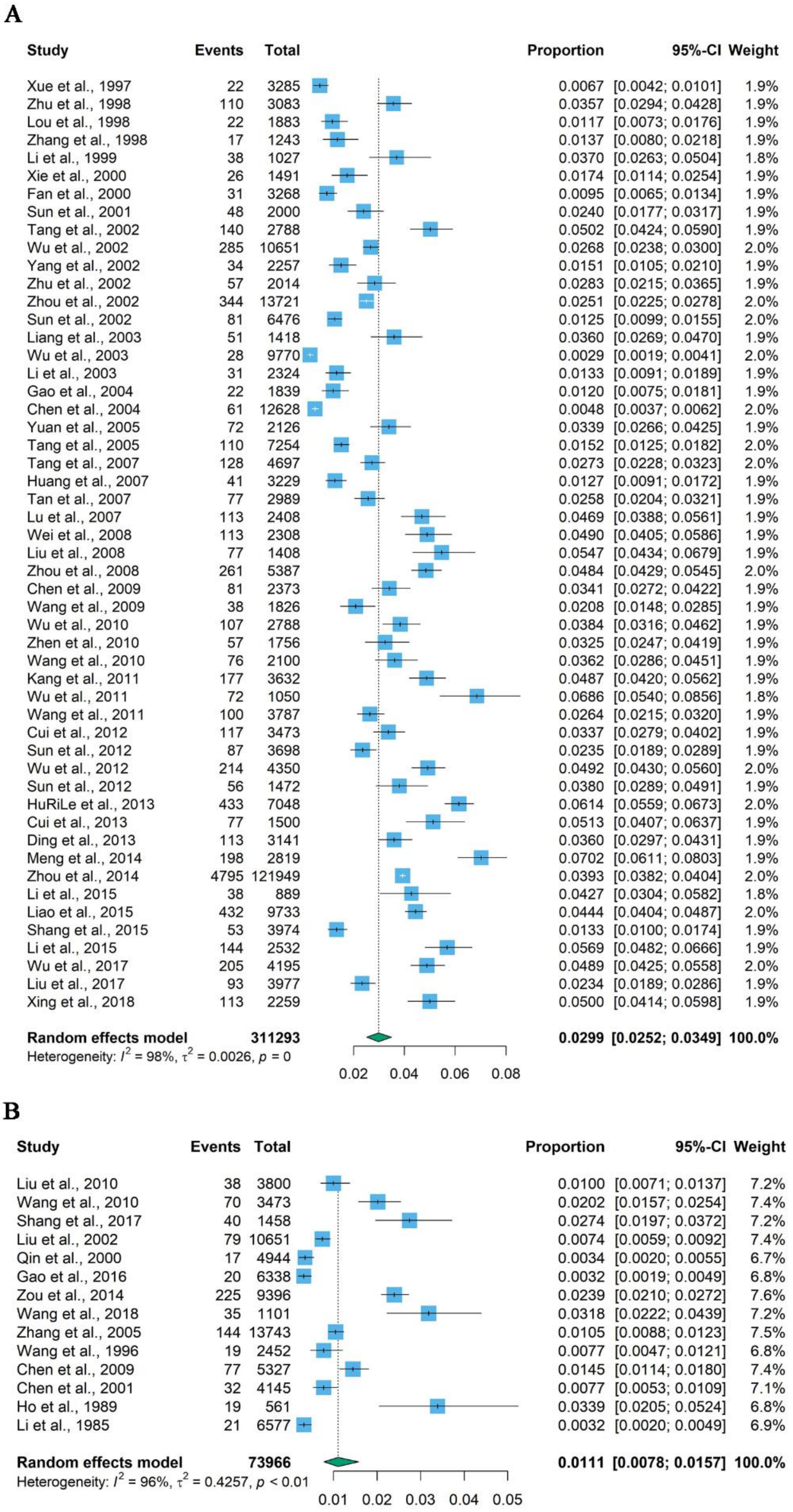

Figure S1. Pooled prevalence of AD (A) and PD (B) in individuals over 60 years old in China by random-effects model. AD, Alzheimer's disease. PD, Parkinson's disease.

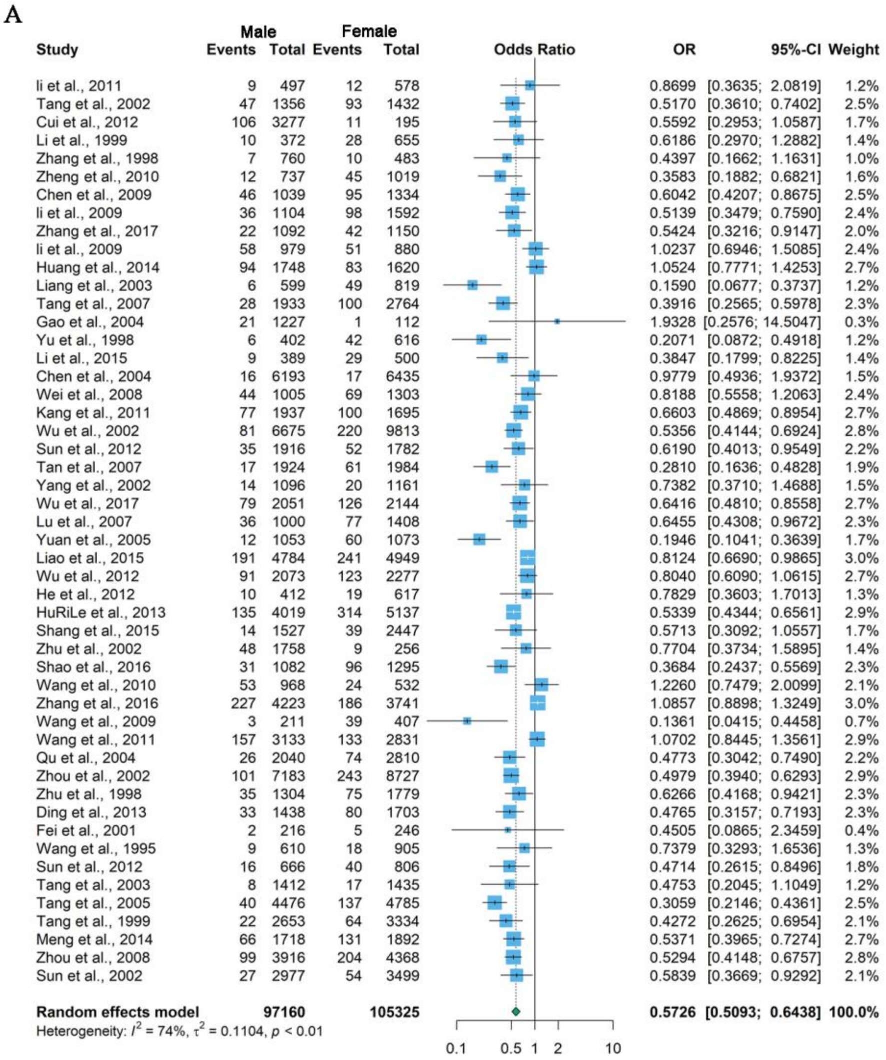

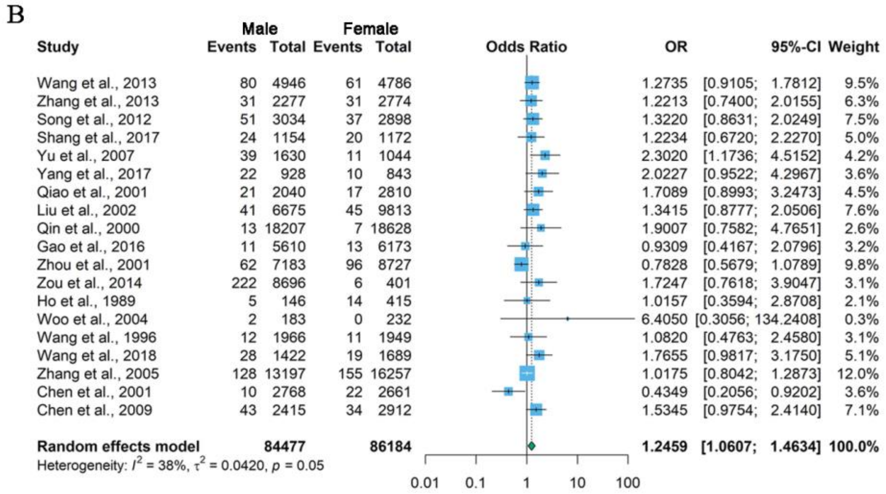

Figure S2. Pooled risk of AD (A) and PD (B) in males compared to females in China by random-effects model. AD, Alzheimer's disease. PD, Parkinson's disease.

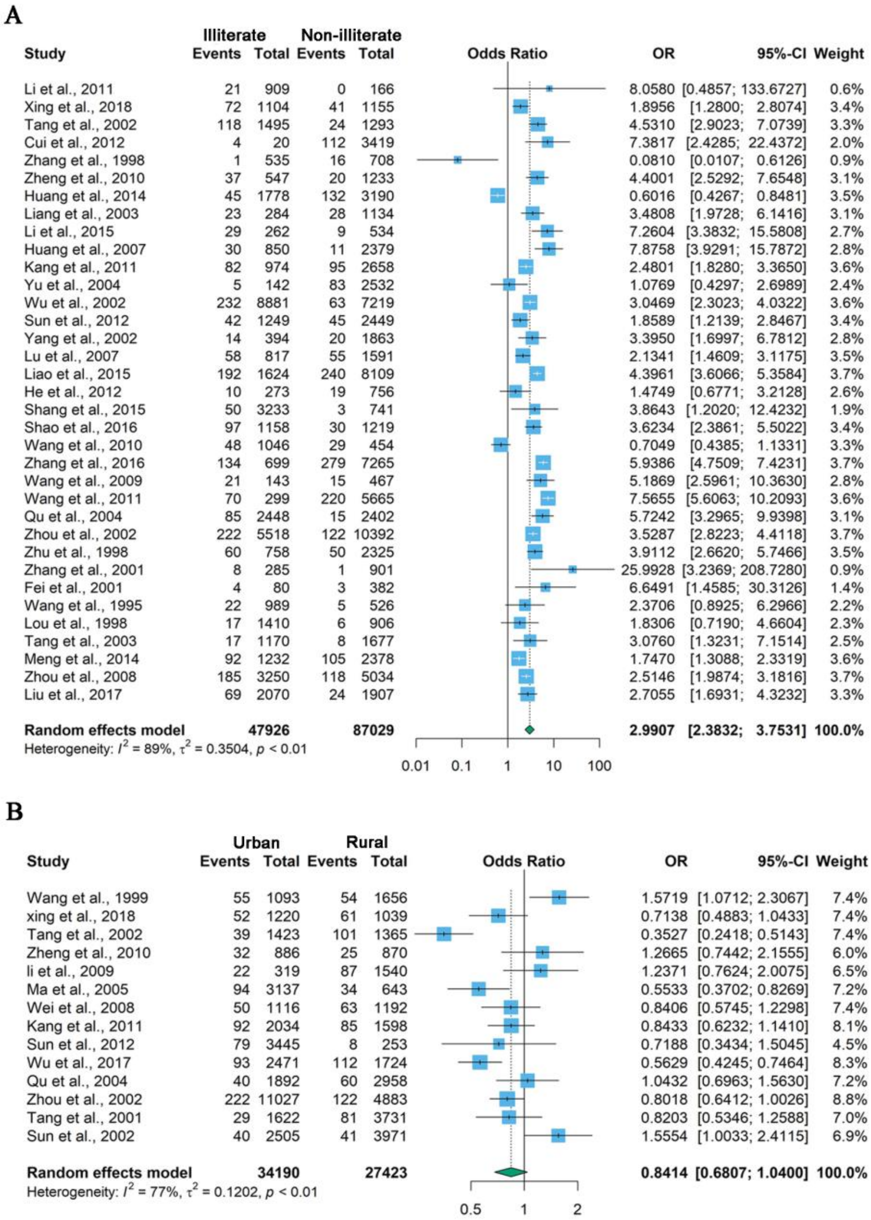

Figure S3. Pooled risk of AD in illiterate (A) and urban (B) compared to their respective counterpart (non-illiterate and rural) by random-effects model. AD, Alzheimer’s disease.
